# Concomitant hepatic tuberculosis and hepatocellular carcinoma: a case report and review of the literature

**DOI:** 10.1186/s12893-020-01021-1

**Published:** 2021-01-02

**Authors:** Hind S. Alsaif, Ali Hassan, Osamah Refai, Khaled Awary, Haitham Kussaibi, Mona H. Ismail, Ibrahim Alghnimi

**Affiliations:** 1 Department of Radiology, King Fahd Hospital of the University, Imam Abdulrahman Bin Faisal University, Al-Khobar, Saudi Arabia; 2 Department of Pathology, King Fahd Hospital of the University, Imam Abdulrahman Bin Faisal University, Al-Khobar, Saudi Arabia; 3 Department of Internal Medicine, King Fahd Hospital of the University, Imam Abdulrahman Bin Faisal University, Al-Khobar, Saudi Arabia; 4 Department of Radiology, Salmaniya Medical Complex, Manama, Bahrain

**Keywords:** Hepatocellular carcinoma, Epatic tuberculosis, Neoplasms, Case reports

## Abstract

**Background:**

Hepatocellular carcinoma (HCC) is the most common primary liver malignancy that is strongly associated with chronic liver disease. Isolated hepatic tuberculosis is an uncommon type of tuberculosis. Concomitant occurrence of both conditions is extremely rare.

**Case presentation:**

We report the case of a 47-year-old man who presented with fever and abdominal pain for 3 months prior to presentation. He reported a history of anorexia and significant weight loss. Abdominal examination revealed a tender, enlarged liver. Abdominal computed tomography (CT) demonstrated a solid heterogeneous hepatic mass with peripheral arterial enhancement, but no venous washout, conferring a radiological impression of suspected cholangiocarcinoma. However, a CT-guided biopsy of the lesion resulted in the diagnosis of concomitant HCC and isolated hepatic tuberculosis.

**Conclusion:**

A rapid increase in tumor size should draw attention to the possibility of a concomitant infectious process. Clinicians must have a high index of suspicion for tuberculosis, especially in patients from endemic areas, in order to initiate early and proper treatment.

## Background

Hepatocellular carcinoma (HCC) is a primary liver malignant tumor that is a leading cause of cancer-related mortality worldwide [1]. It often develops in the setting of chronic liver disease, and it is estimated that more than 70% of cases are related to chronic infection from hepatitis B or C viruses [2]. It is very unusual for HCC to arise in a patient with a non-cirrhotic liver in the absence of risk factors [3].

Tuberculosis is a significant disease worldwide. Abdominal tuberculosis occurs in 5% of all cases of tuberculosis [4]. However, isolated hepatic tuberculosis is a rare form of extrapulmonary tuberculosis. An accumulating body of evidence has indicated the role of infectious agents in oncogenesis. Several Nobel prizes have been awarded to scientists for their work on the relationship between infectious agents and cancer [5]. It is reported that up to 20% of all cancers worldwide are linked to infectious agents [6].

Although the association between tuberculosis and malignancies was first described approximately 200 years ago [7], the co-existence of hepatic tuberculosis and HCC has been reported in a few cases. Herein, we report a middle-aged man diagnosed with HCC and isolated tuberculosis.

## Case presentation

A 47-year-old Indian man presented to the emergency department with a history of fever and abdominal pain for 3 months. The fever was low-grade, intermittent, relieved by antipyretics, and not associated with chills or rigor. Abdominal pain was experienced in the right upper quadrant, sharp in character, and not related to meal consumption. He also reported a cough, anorexia, and unintentional weight loss of 10 kg during the same period. There was no history of vomiting, altered bowel motion, or changes in stool or urine color. A review of other systems revealed unremarkable findings. His medical history was remarkable for diabetes mellitus, which was treated with an oral antidiabetic agent. He had no history of having undergone surgeries previously. He reported no tobacco smoking, alcohol consumption, or intravenous drug abuse. Notably, he reported no recent travel or consumption of unpasteurized dairy products.

On presentation, he was not icteric or pale. He had a pulse rate of 119 bpm, and his other vital signs were within normal limits. Abdominal examination revealed a tender, enlarged liver with a span of 15 cm, and the spleen was palpable 2 cm below the left costal margin. The results of the laboratory studies are summarized in Table 1.

**Table 1:** Summary of the results of laboratory findings

Laboratory investigation	Unit	Result	Reference range
Hemoglobin	g/dL	7.4	13.0–18.0
White blood cell	1000/mL	26.8	4.0–11.0
Platelet	1000/mL	639	140–450
Erythrocyte sedimentation rate	mm/hr	119	0–20
C-reactive protein	mg/dL	24.1	0.05–3.0
Total bilirubin	mg/dL	1.1	0.2–1.2
Albumin	g/dL	2.5	3.4–5.0
Alkaline phosphatase	U/L	596	46–116
Gamma-glutamyltransferase	U/L	294	15–85
Alanine transferase	U/L	124	14–63
Aspartate transferase	U/L	41	15–37
Blood urea nitrogen	mg/dL	8	7–18
Creatinine	mg/dL	1.06	0.7–1.3
Sodium	mEq/L	135	136–145
Potassium	mEq/L	3.4	3.5–5.1
Chloride	mEq/L	98	98–107
Hepatitis B core antibody		Non-reactive	Non-reactive
Hepatitis B surface antibody		Non-reactive	Non-reactive
Hepatitis C virus antibody		Non-reactive	Non-reactive
Carbohydrate antigen 19–9	U/mL	110.7	0–37
Carcinoembryonic antigen	U/mL	1.9	0–3.0
Alpha fetoprotein	ng/mL	35,297	10–15

**Fig. 1:**
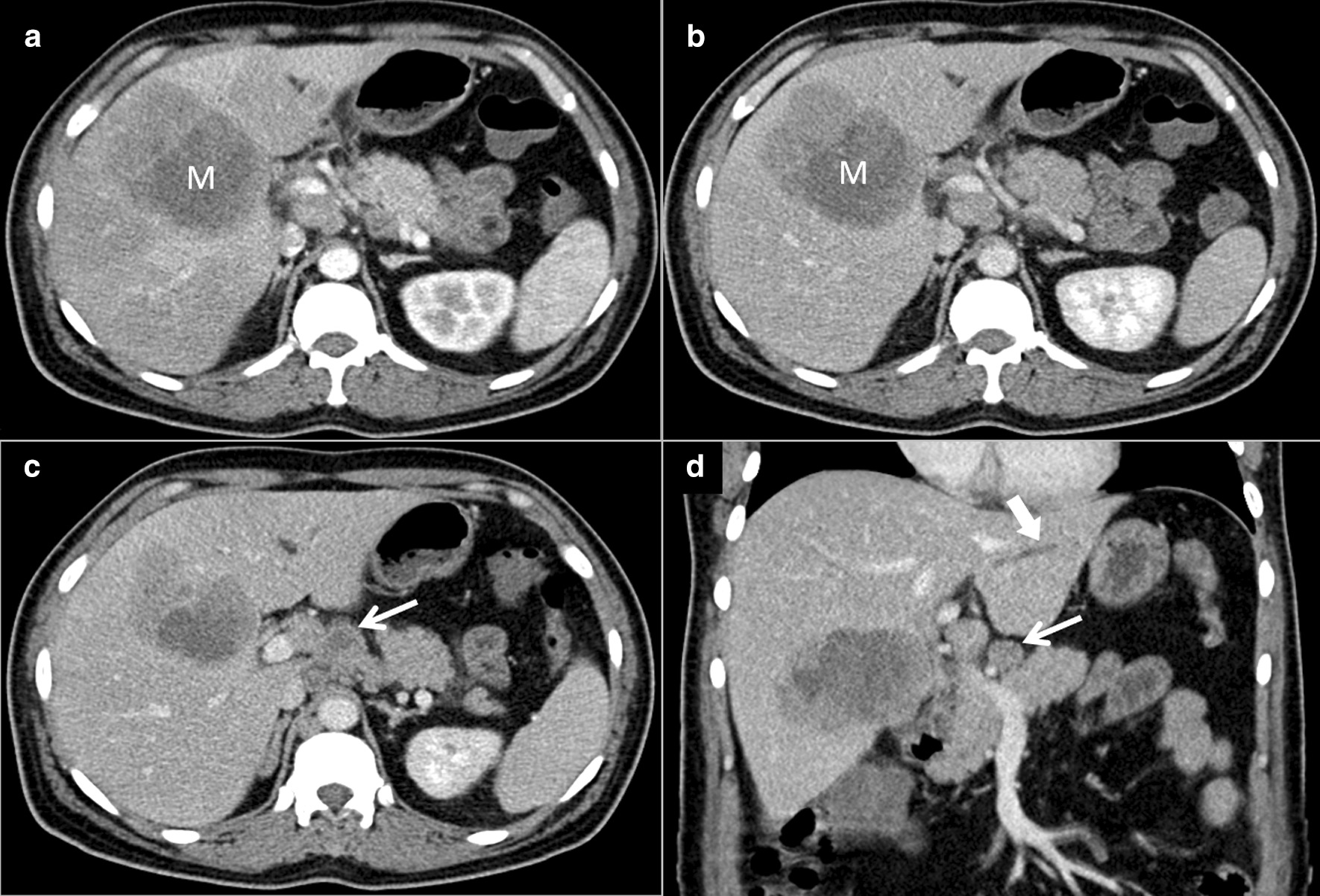
Contrast-enhanced abdominal computed tomography images acquired on initial presentation (**a** axial image in the arterial phase; **b**, **c** axial images in the venous phase; and **d** coronal image in the venous phase) demonstrating a heterogeneous mass (M) with internal hypodensity in segment V (**a** and **b**). Necrotic lymph nodes (thin arrows in **c** and **d**) and mild biliary dilatation (thick arrow in **d**) were noted

Abdominal ultrasonography revealed a solid lesion with heterogeneous echogenicity in the right lobe of the liver in a normal liver background. The mass was further characterized by dedicated contrast-enhanced computed tomography (CT) of the liver in four phases (non-contrast, arterial, portovenous, and delayed phase), which revealed a heterogeneous solid mass measuring 8.5 cm × 6.5 cm × 5.2 cm with peripheral arterial enhancement, internal hypodensity, and no venous or delayed washout. In addition, multiple small satellite lesions were observed throughout the liver. Multiple enlarged lymph nodes were found in the portohepatic, para-aortic, and retrocaval groups with no evidence of thoracic metastases. Dilatation of the left lobe biliary radicals was also observed. Considering that venous washout was not observed, which is a hallmark for the diagnosis of HCC, the radiological impression was of suspected cholangiocarcinoma (Fig. 1).

**Fig. 2:**
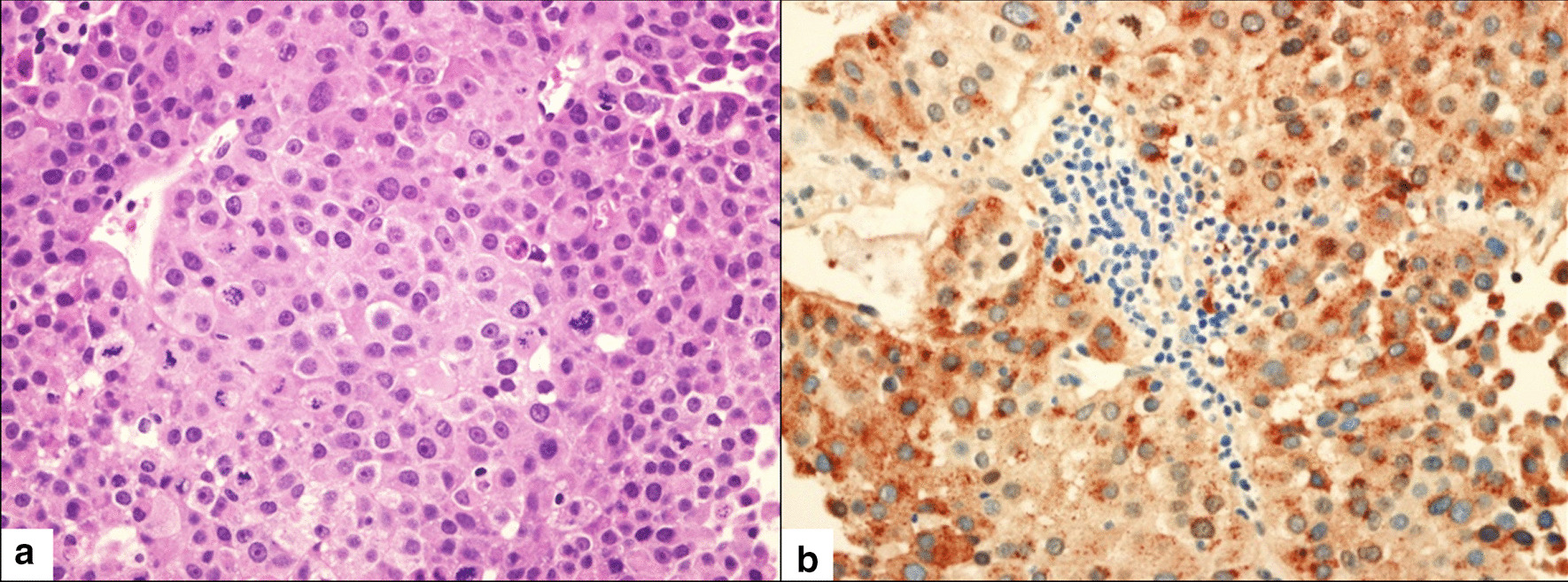
Microscopic view (H&E stain, × 40) showed moderate-to-prominent cellular and nuclear pleomorphism and hyperchromatism along with frequent abnormal mitoses and apoptotic bodies (**a**). The immunohistochemistry view demonstrated the expression of alpha-fetoprotein (**b**). H&E, hematoxylin and eosin

The patient continued to experience multiple episodes of fever during his hospital stay. Percutaneous transhepatic biliary drainage was then performed, as the patient was presumed to have cholangitis due to an obstructive lesion. Moreover, the patient underwent an ultrasound-guided biopsy of the liver lesion, and histopathological examination revealed a poorly differentiated HCC. The tumor cells showed immunohistochemical positivity for CK19, CK8, CK7, AFP, and pCEA and negativity for CK20, CD10, and HepPar1 (Fig. 2).

**Fig. 3:**
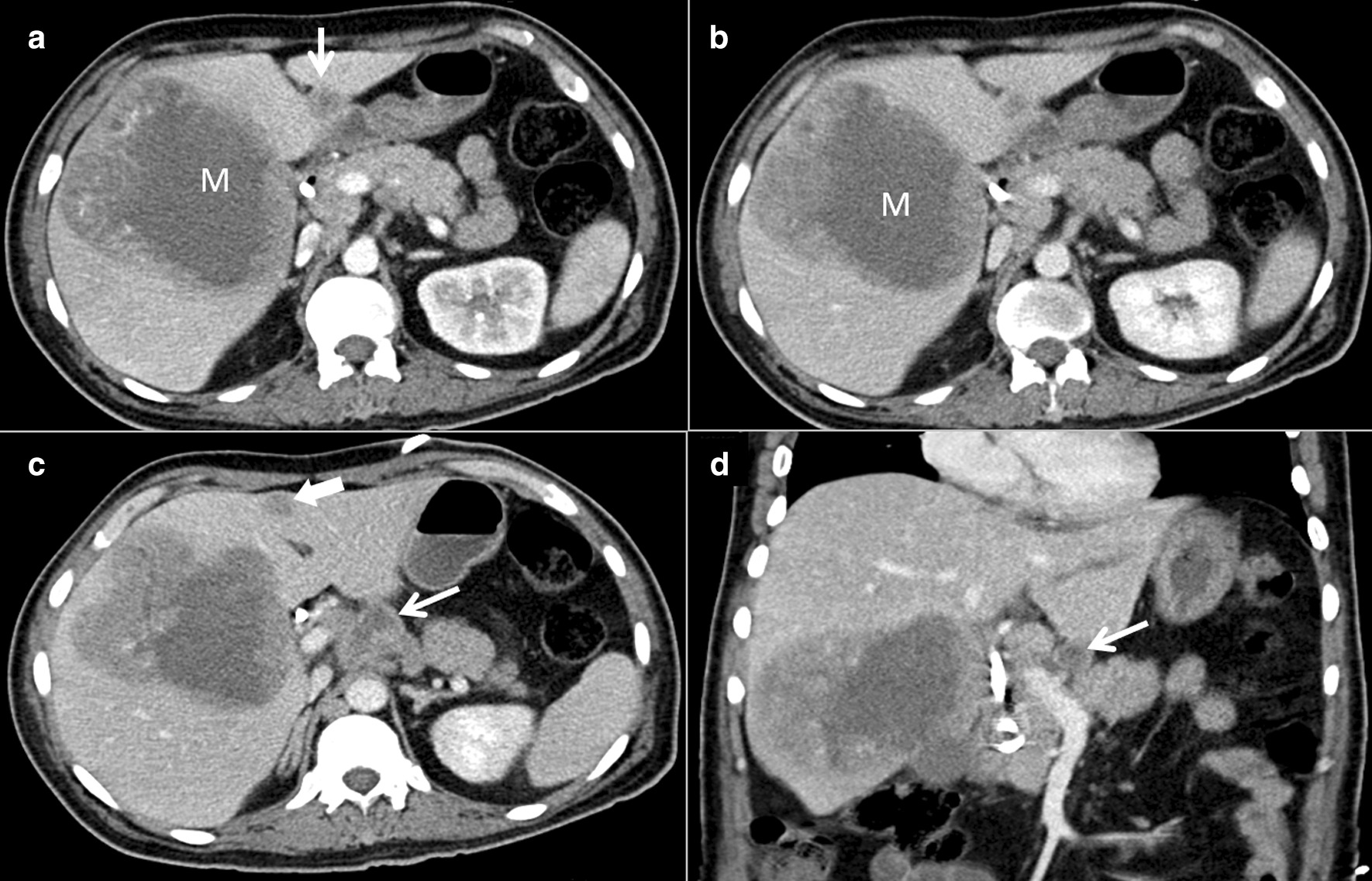
Contrast-enhanced abdominal computed tomography images acquired 1 month following the initial presentation (**a** axial image in the arterial phase; **b**, **c** axial images in the venous phase; and **d** coronal image in the venous phase) demonstrating an interval increase in the size of the mass lesion (M) with larger central hypodensities (**a** and **b)**. Necrotic lymph nodes (thin arrows in **c** and **d**) were observed. A new nodule in the left liver lobe (thick arrow in **c**) and interval increase in the size of the nodule at the falciform ligament (arrow in **a**) were noted

The patient received intravenous metronidazole (500 mg every 8 h) and imipenem (500 mg every 6 h). Intravenous fluconazole (400 mg every 24 h) was also initiated after the biliary culture exhibited growth of *Candida parapsilosis* and *C. tropicalis*. However, the patient did not show any clinical improvement. Abdominal ultrasonography was repeated, which highlighted the progression of the previously identified lesion. Subsequently, abdominal CT revealed that the mass had markedly increased in size compared to that on the initial CT performed 33 days prior, as it measured 11.3 cm × 10.6 cm × 10.6 cm with a larger central hypodense component (Fig. 3). A co-existing infectious process was suspected because of the rapid increase in the size of the lesion. The tissue obtained from the biopsy was sent for routine bacterial culture, acid-fast bacilli smear, and polymerase chain reaction (PCR) testing. The PCR revealed *Mycobacterium tuberculosis* despite the negative acid-fast bacilli smear result.

Subsequently, antitubercular therapy comprising rifampicin (600 mg/day), isoniazid (300 mg/day), ethambutol (1.2 g/day), and levofloxacin (500 mg/day) was initiated. Over the following days, a significant clinical improvement was evident with resolution of the fever. On discharge, the patient was asymptomatic, and antitubercular treatment was continued. Surgical resection was not planned because the tumor was locally advanced with possible lymph node involvement. The patient decided to continue further management in his home country since he was not medically insured.

## Discussion

We report an exceedingly rare case of primary liver malignancy co-existing with isolated hepatic tuberculosis. Only five cases involving conditions similar to this condition have been reported previously. The first case was reported by Wagner in 1861, who discovered HCC and tuberculosis in two separate lesions during autopsy examination [8] (Table 2).

**Table 2:** Review of cases of co-existing hepatic tuberculosis and hepatocellular carcinoma

No.	Year	Author	Age	Gender	Medical history	Clinical presentation	HCC and TB lesions
1	1861	Wagner	NA	NA	NA	Autopsy finding	Different lesions
2	1983	Essop	70	Male	Liver cirrhosis	Vomiting Abdominal pain	Same lesion
3	1994	Yano	72	Female	Diabetes mellitus Hypertension Pleurisy	Asymptomatic	Same lesion
4	2012	Limaiem	73	Female	Hypertension Hepatitis C	Abdominal pain	Same lesion
5	2019	Shah	70	Male	Diabetes mellitus Hypertension Hepatitis C	Decreased appetite Weight loss	Same lesion
6	Current case		47	Male	Diabetes mellitus	Fever Abdominal pain	Same lesion

*TB* tuberculosis, *HCC* hepatocellular carcinoma, *NA* not available

The association between tuberculosis and malignancy may be merely incidental, as both conditions are common. However, there is a growing body of evidence suggesting that mycobacterial infection may play a role in carcinogenesis [9]. For example, *M. tuberculosis* has been shown to have the ability to induce DNA damage via the production of reactive oxygen species [10]. Moreover, the organism may exhibit increased expression of BCL2, which has anti-apoptotic activity [11]. An experimental study has shown that the purified protein derivative of tuberculin can upregulate the expression of vascular endothelial growth factor in lymphocytes, which has significant angiogenic and mitogenic properties [12]. Lastly, it is well known that chronic inflammation, as in mycobacterial infection, is a potential factor in the development of cancers and metastatic spread [13]. Gastric lymphoma is a well-established example in which chronic inflammation, as induced by *Helicobacter pylori* infection, has a causal link with carcinogenesis [14].

The diagnosis of HCC and hepatic tuberculosis was not straightforward in the present case. For example, the radiological findings of hepatic lesion were not typical of HCC. The presence of arterial enhancement in the hepatic lesion and subsequent washout in the portal or delayed phases are considered hallmark imaging features of HCC [15]. However, an atypical appearance of HCC is not uncommon and is reported in approximately 40% of all cases [16]. The presentation of tuberculosis was also atypical in the present case since the chest X-ray findings were normal and the acid-fast bacilli smear result was negative. This case was a useful example of the role of PCR as a rapid and reliable tool for the diagnosis and management of tuberculosis [17].

The median doubling time of hepatocellular lesions is 117 days [18]. In the present case, the volume of the lesion nearly doubled within 27 days, as estimated mathematically [19], which was a rapid increase and led us to suspect the presence of a co-existing infection.

## Conclusion

A rapid increase in tumor size should draw attention to the possibility of a concomitant infectious processes. Clinicians must have a high index of suspicion for tuberculosis, especially in patients from endemic areas, in order to initiate early and proper treatment. Further work is needed to better understand the role of tuberculosis in malignancies.

## Data Availability

Not applicable.

## Abbreviations

CT: Computed tomography
HCC: Hepatocellular carcinoma
PCR: Polymerase chain reaction
